# Positive Gossip Fuels Creativity: The Roles of Cognitive Crafting and Risk Taking

**DOI:** 10.3390/bs15060727

**Published:** 2025-05-24

**Authors:** Sanji Qing, Wenbing Wu, Ying Ma, Ya Wang

**Affiliations:** School of Economics and Management, Beijing Jiaotong University, Beijing 100044, China; 20113082@bjtu.edu.cn (S.Q.); wbwu@bjtu.edu.cn (W.W.); 21113069@bjtu.edu.cn (Y.W.)

**Keywords:** positive workplace gossip, promotion-oriented cognitive crafting, risk-taking behavior, internal locus of control, creativity

## Abstract

This study, based on regulatory focus theory and internal locus of control theory, constructs a moderated mediation model to explore how perceived positive workplace gossip indirectly affects employee creativity through promotion-oriented cognitive crafting and risk-taking behavior. Through the analysis of four-wave, two-source survey data from 463 employees, this study found that perceived positive gossip can stimulate promotion-oriented cognitive crafting in the gossiped-about employees, which in turn promotes risk-taking behavior and ultimately enhances creativity. Furthermore, internal locus of control plays a significant moderating role in this mechanism. The gossiped-about employees with a high internal locus of control are more inclined to respond positively when faced with positive gossip, exhibiting higher promotion-oriented cognitive crafting and risk-taking behavior. Overall, this research advances the understanding of positive gossip’s functional consequences and offers practical insights for fostering organizational creativity.

## 1. Introduction

Gossip is a common topic of conversation in interpersonal interactions (Lian et al., 2023; Cheng et al., 2023; Litman & Pezzo, 2005; Noon & Delbridge, 1993; Foster, 2004) and an important part of workplace culture (Dunbar, 2004; Robbins & Karan, 2020; T. Grosser et al., 2012), with over 90% of employees participating in it (T. J. Grosser et al., 2010). Workplace gossip refers to informal and evaluative conversations among a few people about others who are absent (Kurland & Pelled, 2000; Brady et al., 2017). Based on the nature of the gossip content, workplace gossip is usually divided into negative and positive (Xie et al., 2020). Perhaps because people are more sensitive to negative news (Baumeister et al., 2001; Wang et al., 2025), much of the extant research on workplace gossip has focused on its negative forms (Gao et al., 2024), lacking a sufficient exploration of positive workplace gossip. Scholars believe that negative workplace gossip, as a form of workplace “cold violence,” spreads derogatory information, which intensifies distrust among employees, causes emotional exhaustion, reduces organizational citizenship behavior, and leads to increased knowledge hiding, social avoidance, and turnover, as well as decreased job satisfaction and well-being (Khan et al., 2022, 2023; Babalola et al., 2019; Bai et al., 2020; Wu et al., 2018; Yao et al., 2020; Martinescu et al., 2021; Xie et al., 2020). However, an exclusive focus on these negative aspects offers an incomplete understanding. Building on the recognition that gossip can also be positive, Ellwardt et al. (2012) conceptualized positive workplace gossip as positive evaluations discussed about absent employees, such as defending them or praising their certain behaviors. While foundational studies like Ellwardt et al. (2012) shed light on the functions of positive gossip for gossipers and recipients (e.g., information gathering, social bonding), the specific impact of *perceived* positive gossip on the *target* employee’s subsequent cognitions, behaviors, and particularly complex outcomes like creativity remains less explored. This positive feedback may motivate employees to perform better in subsequent work and thus exhibit higher creativity.

In terms of the actors involved, current research mainly focuses on three categories: the gossiper, the gossip recipient, and the person being gossiped about (Dores Cruz et al., 2019, 2021; Martinescu et al., 2014; Kurland & Pelled, 2000; Lee & Barnes, 2021). The gossiper often fulfills social needs or status pursuits by spreading gossip, thereby gaining control over information and influence while enhancing their presence within the team (Beersma & Van Kleef, 2011). In contrast, gossip recipients gain information and emotional support by participating in gossip exchanges, thus deepening their understanding of organizational dynamics (Ellwardt et al., 2012). However, exposure to negative gossip can elicit adverse emotions and misunderstandings, weakening their trust and commitment to colleagues and the organization (Tan et al., 2021; Dores Cruz et al., 2021). While existing research has deepened our understanding of gossipers and recipients, attention to the specific group of the gossiped-about employees is still relatively insufficient. Although the gossiped-about employees are often the subject of discussion when absent, they can often perceive the occurrence of positive gossip through the unusual behavior of others (Ellwardt et al., 2012) and make cognitive adjustments and behavioral responses based on the information obtained. However, little is known about the specific psychological mechanisms and behavioral pathways triggered within targets upon perceiving positive gossip. If we ignore the internal response patterns and decision-making processes of this subject, our understanding of gossip as an organizational interaction phenomenon will always be incomplete.

**Figure 1:**
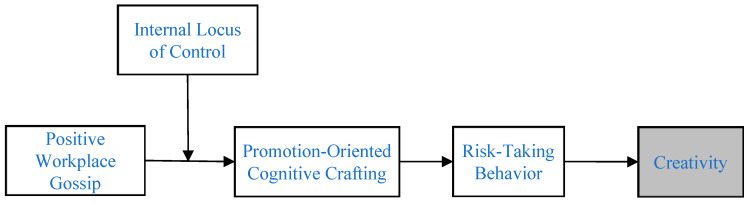
This is a theoretical model. Note. The shaded variable is rated by leaders, and the unshaded variables are rated by the target employees.

To understand the psychology and behavior of the gossiped-about employees in response to perceived positive gossip, we must consider their response mechanisms to external positive information. In addition, individuals exposed to the same environment may exhibit different psychological and behavioral responses (Kahn et al., 1992). To better explain this response mechanism and individual differences, this study integrates regulatory focus theory (Higgins, 1997) and locus of control theory (Rotter, 1966) to construct a moderated mediation model. Regulatory focus theory suggests that, when individuals perceive external positive evaluations, it can activate a promotion orientation (Higgins & Cornwell, 2016; Higgins, 1998; Brockner & Higgins, 2001; Hamstra et al., 2014; Lanaj et al., 2012), prompting them to respond to workplace situations in a way that seeks growth and self-improvement, viewing positive feedback as an opportunity for expansion, and tending to strengthen their image by actively interacting with colleagues, sharing information, and seeking cooperation. Therefore, we selected promotion-oriented cognitive crafting as the first mediator because it represents a key cognitive mechanism through which individuals proactively reframe their work tasks and environment towards growth and achievement goals (Wrzesniewski & Dutton, 2001), directly reflecting the striving associated with an activated promotion focus triggered by positive social feedback. At the same time, the locus of control theory emphasizes an individual’s sense of control over the outcomes of their behavior. Individuals with a high internal locus of control are more likely to interpret positive gossip as recognition of their efforts and abilities, thus stimulating more positive behavior; while individuals with a low internal locus of control may attribute it to luck or environmental factors and are less likely to actively utilize this situation (Hsiao et al., 2016). Based on this, this study proposes that positive workplace gossip may stimulate promotion-oriented cognitive crafting in the gossiped-about employees, which, by fostering an optimistic and opportunity-focused mindset, logically encourages them to take more risk-taking behaviors (such as trying new methods or challenging complex tasks)—our second chosen mediator, crucial for breaking routines and generating novel ideas (George & Zhou, 2001), ultimately promoting the improvement of their creativity. Furthermore, we selected internal locus of control as the moderator because this individual difference directly addresses why some individuals might be more or less responsive to the positive gossip signal and more or less likely to translate it into proactive crafting and risk taking, based on their inherent beliefs about personal agency and control over outcomes (Rotter, 1966). The gossiped-about employees with a high internal locus of control are hypothesized to be more likely to actively adjust their cognition and behavior, transforming the impact of positive gossip into a driving force for enhancing creativity. In contrast, those with a low internal locus of control may be less likely to utilize this positive feedback. Therefore, this study aims to explore how perceived positive workplace gossip, synergistically explained by regulatory focus theory and the locus of control, influences creativity through the sequential mediation of promotion-oriented cognitive crafting and risk-taking behavior, with internal locus of control moderating this indirect pathway. Figure 1 depicts the research model.

This study aims to make theoretical contributions in the following three aspects. First, this study contributes to the gossip literature by building upon foundational work (e.g., Ellwardt et al., 2012) and extending it through exploring the impact mechanism of positive workplace gossip on the creativity of the gossiped-about employees, enriching the related research on workplace gossip. Specifically, this research, by focusing on the target’s perspective of perceived positive gossip, reveals how gossip, as a social interaction phenomenon, can foster employee creativity through specific cognitive and behavioral pathways, thus complementing the previous literature that focused more on negative workplace gossip or on the experiences of gossipers/recipients. Second, from the perspective of the gossiped-about employees, this study explores the sequential mediation pathway of promotion-oriented cognitive crafting and risk-taking behavior, deeply revealing the potential cognitive crafting and behavioral response processes of the gossiped-about employees when facing positive workplace gossip, thereby significantly expanding the research on the impact of workplace gossip on the targets by illuminating how this effect unfolds. This specific mechanism represents a key theoretical advancement. Finally, this study also explores boundary conditions, specifically the moderating role of internal locus of control in the context of perceived positive workplace gossip. This study found that employees with a high internal locus of control are more inclined to transform positive workplace gossip into a driving force for enhancing creativity through promotion-oriented cognitive crafting and risk-taking behavior. In contrast, employees with a low internal locus of control demonstrate a lower willingness to actively transform in this process. This finding provides a more nuanced perspective for understanding the boundary conditions of gossip’s impact and also provides new ideas on how to better stimulate employee creativity by considering individual differences.

## 2. Theory and Hypotheses

### 2.1. Perceived Positive Workplace Gossip and Promotion-Oriented Cognitive Crafting

Perceived positive workplace gossip refers to the psychological process in which the gossiped-about employees indirectly perceive, through informal channels (such as conversations among colleagues, and changes in others’ behavior), that they are the subject of positive evaluations among colleagues, and form a subjective cognition and understanding of this positive evaluation (T. Grosser et al., 2012; Wu et al., 2018). This positive commentary usually involves the excellent performance, positive qualities, or praiseworthy behaviors of the gossiped-about employees. Although these positive comments are not directly conveyed to the individual being evaluated, once they become aware that they are the subject of positive gossip, it often exerts a positive impact on their cognition and behavior. When employees perceive that they are being talked about in a positive way by others, they usually experience feelings of recognition, respect, and appreciation (Dunbar, 2004). This positive feedback can effectively enhance an individual’s self-esteem and self-efficacy (Bandura, 1997), prompting them to form a higher sense of recognition of their abilities and values. This positive enhancement of emotion and cognition not only increases employees’ confidence but may also stimulate positive cognitive responses, such as stronger growth motivation and ambition.

Regulatory focus theory (Higgins, 1997; Brockner & Higgins, 2001) suggests that positive external factors can stimulate an individual’s promotion-oriented motivation. Individuals with a promotion orientation tend to focus on growth, achievement, and aspirations, pursuing positive outcomes and their ideal self. They are more willing to accept challenges and seek innovation and breakthroughs to achieve self-improvement (Gino & Margolis, 2011; Sassenberg et al., 2014). When employees perceive that they are being talked about positively by others, this external positive feedback may trigger their promotion-oriented cognitive crafting, making them more focused on how to leverage their strengths, set higher goals, and achieve greater success.

Specifically, positive workplace gossip makes the gossiped-about employees feel recognized and appreciated by their colleagues. This social recognition prompts them to re-evaluate their abilities and potential (Taylor & Brown, 1988). To match the positive expectations that others have of them, the gossiped-about employees may engage in promotion-oriented cognitive crafting, that is, adjusting their mental patterns to focus more on opportunities and positive possibilities (Higgins, 1997). This cognitive crafting can not only further enhance their growth motivation but may also prompt them to set higher career goals, actively seek development opportunities, and thus improve work performance.

**Hypothesis** **1.** *Positive workplace gossip is positively related to targets’ promotion-oriented cognitive crafting.*

### 2.2. Promotion-Oriented Cognitive Crafting and Risk-Taking Behavior

Promotion-oriented cognitive crafting involves actively framing work tasks and goals in terms of growth, achievement, and aspirations, thereby stimulating stronger growth motivation and a desire for self-improvement in individuals. In this mental state, individuals pay more attention to achieving their ideal self, actively seeking growth opportunities, and thus tend to accept challenges and go beyond their comfort zone (Higgins, 1997). In the workplace, this cognitive tendency manifests behaviorally, making employees more inclined to take risks (Gino & Margolis, 2011) and explore new possibilities to achieve their career goals.

Risk-taking behavior in the workplace refers to employees’ willingness to take risks—engaging in actions with uncertain outcomes—in uncertain situations, trying new methods, strategies, or innovations to obtain greater rewards or promote self-development (Sitkin & Pablo, 1992). Individuals in a promotion-oriented cognitive state do not merely feel motivated; they actively reappraise situations. They tend to view challenges less as threats and more as opportunities for growth and success rather than threats (Zou et al., 2014). Therefore, promotion-oriented cognitive crafting directly encourages employees to engage in risk-taking behaviors by altering their perception of the potential action, fostering innovation and breakthroughs.

According to regulatory focus theory (Higgins, 1997), an individual’s motivational orientation influences their attitude and behavior towards risk. When employees adopt a promotion orientation through cognitive crafting, they are more willing to explore new possibilities and embrace uncertainty and risks, because the potential gains associated with success are salient and highly valued. This shifts the perceived cost–benefit analysis towards action. This promotion-oriented cognition motivates employees to persist in the face of unknown situations, encouraging them to engage in risk-taking behaviors as an instrumental means in pursuit of ideal outcomes (Crowe & Higgins, 1997).

**Hypothesis** **2.** *Targets’ promotion-oriented cognitive crafting is positively related to risk-taking behavior.*

### 2.3. Risk-Taking Behavior and Creativity

Creativity is defined as the ability of employees to produce novel and practical ideas, solutions, or products in their work (Amabile, 1983, 2018). For organizations, creativity is a key factor for continuous innovation and enhancing competitive advantage (Shalley & Gilson, 2004; Zhou & Shalley, 2003). Achieving creativity often requires that employees are willing to break with conventions and routines and explore new paths to cope with uncertainty and challenges (Oldham & Cummings, 1996).

Risk-taking behavior means that employees actively try new methods and explore new areas when facing uncertainty and potential risks. This behavior not only reflects employees’ growth motivation but also serves as a crucial behavioral engine for fostering creative outcomes (Sitkin & Pablo, 1992). Specifically, risk-taking facilitates the cognitive processes essential for creativity. By continuously testing and challenging existing experiences and assumptions, risk-taking behavior provides employees with diverse and often unexpected knowledge inputs and accumulated experiences, thus laying a solid foundation for creative thinking (Simonton, 2003).

In trying and iterating, risk-taking behavior prompts employees to break away from fixed thinking and examine problems from different perspectives (Dyer et al., 2009). This forces engagement in more divergent thinking—exploring a wider range of possibilities. They may adopt different technical means, tools, or thinking strategies from the past to solve complex problems that the organization faces. Although these attempts may not all be immediately successful, the experience of failure itself can also expand the cognitive framework and enrich creative associations and potential solution combinations (Hirst et al., 2009). Furthermore, navigating uncertainty stimulates the conceptual combination needed to forge new paths. As experience accumulates, employees’ ability to cope with risks and uncertainties improves, providing continuous motivation and richer material for generating novel and feasible ideas. Specifically, when employees dare to take risks, challenge the status quo, and try unconventional ideas, they are more likely to break through inertial thinking, integrate existing knowledge with new information in novel ways, and thus propose innovative solutions. In repeated attempts, this integration often finds unique and valuable innovative paths (Mainemelis, 2010). Therefore, encouraging and supporting risk-taking behavior directly cultivates the cognitive flexibility and diverse inputs that effectively unleash employees’ creativity.

**Hypothesis** **3.** *Targets’ risk-taking behavior is positively related to their creativity.*

### 2.4. The Indirect Effect of Positive Gossip on Employee Creativity

As discussed above, when employees learn that they are being talked about positively among colleagues, they internalize this positive social feedback and tend to examine their growth opportunities and potential from a promotion-oriented perspective (Higgins, 1997; Neubert et al., 2008). This cognitive crafting not only enhances employees’ intrinsic motivation and self-expectations (Ryan & Deci, 2000; Taylor & Brown, 1988) but also makes them more open to future challenges and changes, thus making it easier to take risk-taking behaviors (Sitkin & Pablo, 1992). Risk-taking behavior provides fertile ground for creative thinking and the generation of novel ideas (Amabile, 1983; Zhou & Shalley, 2003). Therefore, positive workplace gossip changes employees’ psychological orientation through promotion-oriented cognitive crafting, motivating them to face the work environment and development opportunities more actively; this positive cognition further promotes employees to take risk-taking behaviors, seeking unique solutions and innovative paths.

**Hypothesis** **4.** *Positive workplace gossip is positively and indirectly related to targets’ creativity through their promotion-oriented cognitive crafting and risk-taking behavior.*

### 2.5. The Moderating Role of Internal Locus of Control

Internal locus of control reflects the degree to which an individual feels in control of the outcomes of their behavior. Individuals with a high internal locus of control generally believe that they can influence external outcomes and achieve goals through their efforts (Rotter, 1966). In the context of positive workplace gossip, internal locus of control may play a key moderating role in how employees internalize this positive feedback and engage in promotion-oriented cognitive crafting. Employees with a high internal locus of control are more inclined to view the positive evaluations of others as recognition of their efforts and abilities. This process of self-attribution makes them more motivated to continue self-improvement and growth in the workplace (Judge et al., 2003). Therefore, when they learn that they are the subject of positive gossip, they will be more active in promotion-oriented cognitive crafting and more willing to focus on opportunities and the achievement of success.

Conversely, individuals with a low internal locus of control, although they may also feel recognized by positive gossip, tend to attribute outcomes to external factors, such as luck or the environment, rather than their controllable efforts and abilities (Spector, 1988). This leads them to internalize the positive feedback to a lesser extent and make fewer positive cognitive adjustments. According to regulatory focus theory (Higgins, 1997), employees with a high internal locus of control exhibit a stronger promotion orientation because they are more willing to transform external positive feedback into a driving force for self-improvement. Therefore, in the context of positive gossip, employees with a high internal locus of control will more actively process their cognitive patterns, focusing on growth and achieving higher goals.

**Hypothesis** **5.** *Targets’ internal locus of control moderates the positive relationship between positive workplace gossip and promotion-oriented cognitive crafting, such that this relationship is stronger when internal locus of control is higher (vs. lower).*

Building upon the proposed mediating pathway, we further integrate locus of control theory (Rotter, 1966) to understand individual differences in the strength of this indirect effect. While perceived positive gossip may activate a promotion focus, the locus of control theory suggests that individuals differ in their perceived ability to influence outcomes through their actions. We argue that the internal locus of control acts as a critical amplifier throughout the serial mediation process.

Specifically, individuals high in internal locus of control, who believe they have personal agency over events, are likely to more effectively translate the positive social cue inherent in perceived positive gossip into proactive promotion-oriented cognitive crafting (Stage 1). They see the positive feedback as a direct result of their capabilities and are thus more motivated to build upon it cognitively. Furthermore, this enhanced proactive mindset fostered by cognitive crafting is more likely to be converted into concrete risk-taking behaviors (Stage 2) among high internal-locus-of-control individuals. Their inherent confidence in their ability to manage challenges and achieve desired outcomes makes them more willing to embrace the uncertainty associated with novel approaches and experimentation, which are central to risk taking (Hsiao et al., 2016).

Conversely, individuals low in internal locus of control may attribute positive gossip to external factors (e.g., luck, others’ kindness) and thus feel less personal impetus to engage deeply in promotion crafting. Even if they do engage in some crafting, their lower belief in personal efficacy may inhibit them from translating that proactive mindset into tangible, potentially risky actions. They might perceive the potential downsides of risk-taking as beyond their control.

Therefore, the internal locus of control is expected to moderate the strength of the entire positive indirect pathway. The positive effect of perceived gossip on creativity, flowing sequentially through promotion-oriented cognitive crafting and risk-taking behavior, should be significantly stronger for individuals with a high internal locus of control compared to those with a low internal locus of control, because a high internal locus of control facilitates more effective translation at both key stages of the mediation chain.

**Hypothesis** **6.** *Targets’ internal locus of control moderates the positive indirect effects of positive workplace gossip on creativity through the serial mediating effect of promotion-oriented cognitive crafting and risk-taking behavior, such that this indirect effect is stronger when the internal locus of control is higher (vs. lower).*

## 3. Method

### 3.1. Participants and Procedures

To comprehensively investigate the impact of positive workplace gossip on employee creativity and enhance the generalizability and practical implications of our findings, this study was conducted across three companies in different industries in China. These three companies represent the technology, manufacturing, and logistics sectors, with each company individually employing approximately 1000 workers, although their organizational cultures and industry characteristics vary. An author, with a broad network across multiple industries, assisted the research team in contacting and obtaining participation permission from these three companies. Data collection was conducted using on-site paper-based questionnaires. In each company, we first collaborated with the human resources department to identify participating employees and their direct supervisors. To ensure robust data pairing and explicitly avoid situations where one leader evaluates multiple employees within this study’s framework, we adopted a strictly enforced one-to-one pairing approach for the Time 4 creativity assessment, where each participating leader evaluated only one pre-assigned employee’s creativity. The purpose, requirements, confidentiality, and anonymity of the survey were emphasized to each participant in the announcement and during the survey process. All participants signed informed consents before conducting the formal survey. We assigned a unique code to each predetermined leader–employee pair for data matching. We distributed questionnaires to 640 initially identified leader–employee pairs across the three companies. Paper questionnaires were distributed and collected centrally at the workplace to ensure a high response rate and data quality.

To avoid common method bias (Podsakoff et al., 2003), we adopted a four-wave, two-source design method and set the time interval for each wave to two weeks (see a similar approach: Nifadkar et al., 2012; Zhang et al., 2017). Data collection commenced in July 2024 and concluded in late August 2024. Specifically, at Time 1 (conducted starting mid-July 2024, around 15 July 2024), employees reported their age, gender, education, organizational tenure, perceived positive workplace gossip about themselves, and internal locus of control. Two weeks later, at Time 2 (conducted starting late July 2024, around 29 July 2024), employees assessed promotion-oriented cognitive crafting. Another two weeks later, at Time 3 (conducted starting mid-August 2024, around 12 August 2024), we measured employees’ risk-taking behavior. Finally, two weeks after Time 3, at Time 4 (conducted starting late August 2024, around 26 August 2024), creativity was reported by their designated leaders.

After deleting the missing data, we finally successfully matched 463 leader–employee pairs (final response rate = 72.34%). The average age of participants was 36.31 years (SD = 7.09), 54.00% were male, 76.46% held a bachelor’s degree or above (SD = 0.89), and their average organizational tenure was 4.97 years (SD = 2.79). The average age of participating leaders (supervisors) was 42.5 years (SD = 3.1), 65.01% were male, over 63.93% held a bachelor’s degree or above, and their average managerial tenure was 8.7 years (SD = 3.5).

### 3.2. Measures

All measuring materials were present in Chinese. The specific items used to measure each construct in this study are presented in Appendix A. Following Brislin’s (1986) standard translation and back-translation procedures, all items were translated from English to Chinese. Unless otherwise noted, all items were rated on a seven-point Likert-type scale (1 = strongly disagree and 7 = strongly agree).

Positive workplace gossip: Positive workplace gossip was assessed by Chandra and Robinson’s (2009) three items. An example item is “In the past six months, others (e.g., coworkers and/or supervisors) communicated positive information about me in the workplace”.

Internal locus of control: We used Hsiao et al.’s (2016) three items to capture the target employees’ internal locus of control. An example item is “I believe that my level of involvement determines the resulting outcome”.

Promotion-oriented cognitive crafting: This variable was measured by Bindl et al.’s (2019) four items. An example item is “I focused my mind on the best parts of my job, while trying to ignore those parts I didn’t like”.

Risk-taking behavior: Neves and Eisenberger’s (2014) four items were used to measure risk-taking behavior. An example item is “I often put myself in a position of risk to help this organization”.

Creativity: Baer and Oldham’s (2006) four items were used to measure creativity. An example item is “This employee suggests many creative ideas that might improve working conditions”.

Control variables: Consistent with similar studies on gossip (Gao et al., 2024; Xie et al., 2024; Martinescu et al., 2021), we also controlled for the target employees’ age, gender, education, and organizational tenure.

### 3.3. Results

Table 1 represents the means, standard deviations, reliabilities, and correlations of all variables. We conducted a series of confirmatory factor analyses for the hypothesized six-factor model and the alternative models. As shown in Table 2, the hypothesized five-factor model fit the data better than other alternative models [χ^2^(125) = 265.93, RMSEA = 0.05, SRMR = 0.03, CFI = 0.97, TLI = 0.97]. Thus, these results supported the discriminant validity of core variables. The results of Harman’s single-factor test showed that the first factor loading was 27.73% (<40%), indicating that there was no serious common method bias in this study (Podsakoff et al., 2003).

To test our hypotheses, we performed regression analyses in Mplus 8.3 (Muthén & Muthén, 1998–2017). Table 3 demonstrates the results of regression analyses for direct effects. Hypothesis 1 posits that positive workplace gossip is positively related to targets’ promotion-oriented cognitive crafting. As we predicted, the results showed that the relationship between positive workplace gossip and promotion-oriented cognitive crafting was significant and positive (β = 0.16, SE = 0.02, *p* < 0.001). Hypothesis 2 assumes that targets’ promotion-oriented cognitive crafting is positively related to risk-taking behavior. In line with our assumption, the relationship between promotion-oriented cognitive crafting and risk-taking behavior was significant and positive (β = 0.43, SE = 0.07, *p* < 0.001). Hypothesis 3 posits that targets’ risk-taking behavior is positively related to their creativity. The results showed that risk-taking behavior is positively associated with creativity (β = 0.38, SE = 0.05, *p* < 0.001). Hence, Hypotheses 1, 2, and 3 were supported.

To examine the mediation effect, moderation effect, and moderated mediation effect, conditional process analyses were performed by using 10,000 bootstrapped samples to place 95% confidence intervals of the estimates. The data were mean-centered prior to testing our model (Aiken & West, 1991). Hypothesis 4 states that positive workplace gossip is positively and indirectly related to targets’ creativity through their promotion-oriented cognitive crafting and risk-taking behavior. Our analyses (in Table 4) revealed that the serial mediating effect on creativity is significant and positive (indirect effect = 0.03, SE = 0.01, 95% CI = [0.02, 0.04]). Thus, Hypothesis 4 was supported.

**Figure 2:**
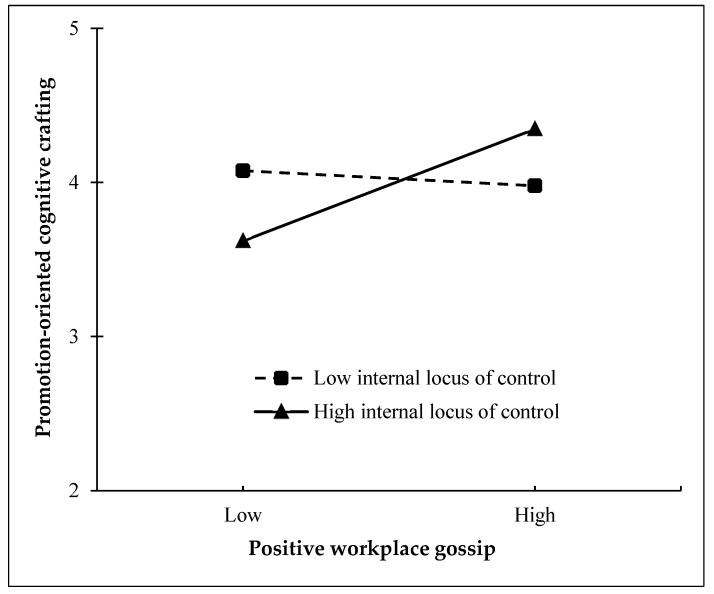
The moderating effect of internal locus of control on the relationship between positive workplace gossip and promotion-oriented cognitive crafting.

Hypothesis 5 focuses on the moderated effect of the targets’ internal locus of control on the relationship between positive workplace gossip and promotion-oriented cognitive crafting. Table 3 displayed that this interaction effect was significant and positive (β = 0.21, SE = 0.03, *p* < 0.001). Furthermore, we plotted the relationship between positive workplace gossip and promotion-oriented cognitive crafting at high (Mean + 1 SD) and low (Mean − 1 SD) levels of internal locus of control (Aiken & West, 1991). Figure 2 demonstrated that the relationship between positive workplace gossip and promotion-oriented cognitive crafting was significant and stronger at a high level of internal locus of control (β = 0.36, SE = 0.03, 95% CI = [0.29, 0.42]), whereas it was non-significant at a low level of internal locus of control (β = −0.05, SE = 0.04, 95% CI = [−0.13, 0.04]). Thus, this finding supported Hypothesis 5.

Hypothesis 6 proposes that targets’ internal locus of control moderates the positive indirect effects of positive workplace gossip on creativity through the serial mediating effect of promotion-oriented cognitive crafting and risk-taking behavior, such that this indirect effect is stronger when internal locus of control is higher (vs. lower). The conditional process analyses (in Table 4) revealed that the positive indirect effect was significantly stronger when the internal locus of control was higher (estimate = 0.06, SE = 0.01, 95% CI = [0.04, 0.09], compared with when the internal locus of control was lower (estimate = −0.01, SE = 0.01, 95% CI = [− 0.02, 0.01]). Moreover, the difference between these two conditional indirect effects at high and low levels was significant (difference = 0.07, SE = 0.02, 95% CI = [0.04, 0.11]). Hence, Hypothesis 6 was supported.

## 4. Discussion

### 4.1. Theoretical Implications

First, this study, based on regulatory focus theory, reveals how positive workplace gossip ultimately promotes the creativity of the gossiped-about employees by influencing their cognition and behavior, thus expanding the application of this theory in explaining how external social situations affect individual cognitive orientations. Although regulatory focus theory (Higgins, 1997; Brockner & Higgins, 2001) has been widely applied in motivation and behavior research, previous studies have mainly focused on internal motivational factors of individuals, with less discussion on how external social situations such as workplace gossip affect individual cognitive crafting. This study, by applying regulatory focus theory, deeply reveals the impact mechanism of positive workplace gossip on employee creativity. Regulatory focus theory divides an individual’s motivational orientation into promotion and prevention focus, the former focusing on ideals and growth, and the latter focusing on safety and responsibility (Higgins, 1997; Wallace & Chen, 2006). This study found that positive workplace gossip, as a positive social message, can activate the promotion focus of the gossiped-about employees in specific situations, prompting them to engage in promotion-oriented cognitive crafting, paying more attention to growth and achievement goals (Neubert et al., 2008). It is worth noting that this study emphasizes the core position of promotion-oriented cognitive crafting in the impact process of positive gossip, revealing how individuals internalize external positive information as a driving force for self-improvement by adjusting their cognitive patterns. This process not only verifies the expectations of regulatory focus theory but also reveals from a micro-level how social situations (positive gossip) affect individual motivation and behavior, providing a new perspective for understanding the formation of employee motivation in organizations.

Second, this study enhances the theoretical foundation of the gossip research area by thoroughly examining the intricate mechanisms of workplace gossip’s impact on individuals from the viewpoint of the gossiped-about employees. Building upon foundational work like Ellwardt et al. (2012), which primarily identified gossip functions for various actors (gossipers, recipients), this study significantly advances understanding by specifying the intra-individual psychological pathway (promotion-oriented cognitive crafting leading to risk-taking behavior) through which perceived positive gossip translates into enhanced creativity for the target. Previous studies have mainly focused on the impact of gossip on the disseminators and recipients, such as the role of gossip on trust, team cohesion, and organizational culture (Kniffin & Sloan Wilson, 2010; Brady et al., 2017), while less attention has been paid to the impact on the gossiped-about employees. This study focuses on the psychological and behavioral changes in the gossiped-about employees, regarding gossip as an important source of social information, and, by examining its impact on individual cognition and behavior, introduces a new perspective to the study of gossip impact pathways. Specifically, this study found that positive gossip makes the gossiped-about employees feel recognized and appreciated by their colleagues, stimulating their promotion-oriented cognitive crafting and risk-taking behavior (T. Grosser et al., 2012). This process emphasizes the key role of the subjective experience and cognitive crafting of the gossiped-about employees in the gossip impact mechanism, revealing that gossip is not only information transmission but also an important influencing factor of individual psychology and social behavior, thus expanding our understanding of gossip impact pathways (Dunbar, 2004). By concentrating on the psychological and behavioral changes in people being gossiped about, we reveal that gossip is not just a vehicle for information distribution but also a significant factor influencing employees’ intrinsic motivation and behavioral performance. This shift in perspective provides a new direction for future gossip research (Michelson & Suchitra Mouly, 2004).

Third, this study reveals the boundary conditions of locus of control in the impact process of positive workplace gossip and deepens the understanding of the mechanism of individual differences in the impact of positive workplace gossip (Rotter, 1966; Spector, 1988). Specifically, we found that employees with a high internal locus of control are more inclined to view the positive evaluations of others as a recognition of their abilities and efforts when faced with positive gossip, more actively engaging in self-attribution, and thus more actively engaging in promotion-oriented cognitive crafting and risk-taking behavior, ultimately enhancing creativity (Judge et al., 2003). Employees with a low internal locus of control may attribute positive gossip to external factors, reducing the stimulation of intrinsic motivation. Meanwhile, this also provides a new theoretical perspective for gossip research, pointing out that the impact of gossip is not universally applicable but is limited by the individual’s internal locus of control.

### 4.2. Practical Implications

Based on the findings of this study, which show that employees who perceive themselves as the subject of positive gossip are more likely to engage in promotion-oriented cognitive crafting and risk-taking behavior, thereby enhancing creativity. Therefore, organizations should not only recognize the potential of positive gossip but actively cultivate an environment where it can thrive. Organizations should actively guide and utilize positive gossip to create a positive work environment and enhance employees’ self-efficacy and innovation capabilities (Bandura, 1997; Higgins, 1997). Specifically, to foster such a culture, managers can lead by example, modeling positive talk by authentically sharing positive comments about employee contributions (ethically), thereby setting a norm that values positive information. Furthermore, they should actively amplify successes by sharing specific examples of achievements and helpful behaviors via multiple channels (meetings, intranets, conversations), focusing on concrete impact to keep any informal talk constructive. Encouraging team activities and projects where employees can foster positive interactions by observing and appreciating colleagues’ strengths also creates fertile ground for such positive informal talk. Additionally, offering training on giving and receiving constructive positive feedback can improve the overall communication climate, indirectly supporting positive dialogue. Crucially, managers must also set clear boundaries by discouraging negative gossip through clear expectations for respectful communication and promptly addressing harmful talk, thus protecting the positive channel’s credibility. Complementing these cultural strategies, formal recognition mechanisms also remain important. Managers can publicly praise employees’ excellent performance through regular recognition meetings, setting up “praise walls” or “shining moments” columns, thereby promoting the formation of a positive feedback culture (T. Grosser et al., 2012). This recognition not only enhances employee confidence and promotes team trust and cooperation but also strengthens promotion-oriented cognition, stimulating employees’ risk-taking behavior and creativity (Dunbar, 2004).

This study also found that internal locus of control plays a significant moderating role in the process through which employees perceive positive gossip and transform it into positive behavior. Therefore, organizations should consider individual differences among employees in practice. For employees with a high internal locus of control, organizations can further stimulate their creative potential by providing more challenging work tasks, greater autonomy, and development opportunities (Judge et al., 2003). For employees with a low internal locus of control, organizations can provide more guidance and support to help build confidence and gradually cultivate a positive perception of their abilities. At the same time, organizations can also enhance employees’ internal locus of control through training and development programs, enabling them to better utilize positive feedback and, in turn, boost overall creativity.

### 4.3. Research Limitations and Future Directions

Although this study adopted a multi-source data collection method, including multiple sources from the gossiped-about employees and their supervisors, to reduce the social desirability bias that may be brought about by single self-reports (Podsakoff et al., 2003), there are still certain limitations. On the one hand, the self-report part of the data may still be affected by social pressure or self-presentation tendencies, leading to limitations in the objectivity of some results. On the other hand, there may be consistency issues between information from different sources, especially in the assessment of subjective feelings and behavioral performance. Therefore, future research can further optimize data collection strategies, such as increasing on-site observations and behavioral records, to enhance the objectivity and reliability of the data. In addition, incorporating a wider range of third-party assessment tools, such as psychological assessments or performance indicators, will also help to comprehensively understand the impact mechanism of positive gossip on employee behavior and creativity.

Second, although this study used a four-wave two-source data collection method, it is still not sufficient to fully establish the causal direction due to the lack of strict time-series analysis (Shadish et al., 2002). Although the multi-timepoint data design offers advantages over traditional cross-sectional studies, the inability to impose stricter time-series control on the order of changes and impact processes for each variable limits causal inference. Therefore, future research could extend the measurement period or increase the number of measurement waves based on the longitudinal design. Additionally, time-series models or quasi-experimental strategies could be employed to more clearly present the dynamic chain of action between positive workplace gossip, promotion-oriented cognitive crafting, risk-taking behavior, and creativity, thereby providing a stronger test and validation of the theoretical model proposed in this study.

Third, this study focused exclusively on the functional consequences of perceived positive workplace gossip for creativity. However, even gossip framed positively might carry unintended negative consequences that were not captured here. For instance, being the subject of positive gossip could potentially create performance pressure, generate envy among peers, or inadvertently highlight in-group/out-group dynamics. Future research should adopt a more nuanced perspective, exploring both the bright and potential dark sides of seemingly positive gossip to provide a more balanced understanding.

Finally, this study was mainly conducted within a Chinese cultural context, which has a strong collectivist character. Chinese culture emphasizes the harmony of interpersonal relationships and collective belonging, which may affect the way that positive workplace gossip is spread and its impact (Hofstede, 1980). In collectivist cultures, employees are more likely to express positive evaluations and prioritize the feelings of others, potentially reducing the motivational effect of positive gossip. Therefore, the generalizability of our findings to other cultural contexts, particularly individualistic Western cultures, warrants caution. However, these findings may not be directly generalizable to cultural contexts that are mainly individualistic. Furthermore, while we explored the moderating role of internal locus of control, this study did not examine potential interactions between individual differences like locus of control and broader contextual factors, such as organizational climate or specific cultural nuances beyond collectivism vs. individualism. Therefore, future research should aim to expand to different cultural contexts, especially in Western countries with predominantly individualistic cultures, to compare the effects of positive workplace gossip across various cultural settings (Hofstede, 1980). By conducting cross-cultural comparative studies, we can better understand the moderating role of cultural background in gossip dissemination and its impact on employee behavior, thereby increasing the general applicability of research conclusions and providing more targeted management recommendations for different cultural contexts.

## 5. Conclusions

This study explored how positive workplace gossip indirectly affects employee creativity through promotion-oriented cognitive crafting and risk-taking behavior, with locus of control playing a significant moderating role in this mechanism. The results showed that employees with a high internal locus of control are more likely to enhance creativity through cognitive crafting and risk-taking behavior when faced with positive gossip. This study enhances understanding of the mechanism of positive workplace gossip but also provides specific practical suggestions for organizational management.

## Figures and Tables

**Table 1:** Descriptive statistics and correlations among study variables.

Variables	Mean	SD	1	2	3	4	5	6	7	8	9
1. Age	36.31	7.09	—								
2. Gender	0.54	0.50	0.01	—							
3. Education	2.98	0.89	0.04	−0.07	—						
4. Tenure	4.97	2.79	0.25 **	0.08	0.01	—					
5. Positive workplace gossip	4.02	0.87	0.08	−0.04	−0.03	−0.03	**0.89**				
6. Internal locus of control	4.28	0.85	0.03	0.05	−0.05	0.01	−0.07	**0.91**			
7. Promotion-oriented cognitive crafting	3.98	0.57	0.05	−0.06	−0.003	0.003	0.29 **	−0.06	**0.85**		
8. Risk-taking behavior	4.04	0.74	0.07	0.01	−0.01	−0.05	0.26 **	−0.08	0.33 **	**0.90**	
9. Creativity	4.07	0.80	0.01	0.01	−0.02	−0.03	0.11 *	0.01	0.21 **	0.35 **	**0.92**

Note. *N* = 463. * *p* < 0.05, ** *p* < 0.01. Gender: 0 = female, 1 = male. Education: 1 = high school diploma or below, 2 = associate’s degree, 3 = bachelor’s degree, and 4 = master’s degree or above. Diagonal elements (in bold) are Cronbach’s alphas.

**Table 2:** Results of confirmatory factor analysis.

CFA Models	*χ^2^*	*df*	CFI	TLI	RMSEA	SRMR
Five-factor model	265.93	125	0.97	0.97	0.05	0.03
Four-factor model (combining positive workplace gossip and internal locus of control)	1183.64	129	0.80	0.77	0.13	0.10
Four-factor model (combining internal locus of control and promotion-oriented cognitive crafting into one factor)	1150.52	129	0.81	0.77	0.131	0.15
Four-factor model (combining promotion-oriented cognitive crafting and risk-taking behavior into one factor)	962.06	129	0.84	0.82	0.118	0.09
Four-factor model (combining risk-taking behavior and creativity into one factor)	1421.20	129	0.76	0.71	0.15	0.10
Three-factor model (combining positive workplace gossip, internal locus of control, and promotion-oriented cognitive crafting into one factor)	1922.84	132	0.67	0.61	0.17	0.14
Three-factor model (combining positive workplace gossip and internal locus of control into one factor; combining promotion-oriented cognitive crafting and risk-taking behavior into one factor)	1879.66	132	0.67	0.62	0.17	0.14
Three-factor model (combining promotion-oriented cognitive crafting, risk-taking behavior, and creativity into one factor)	2105.53	132	0.63	0.57	0.18	0.14
Two-factor model (combining positive workplace gossip and internal locus of control into one factor; combining promotion-oriented cognitive crafting, risk-taking behavior, and creativity into one factor)	3021.90	134	0.46	0.38	0.22	0.17
One-factor model (combining all variables into one factor)	3746.81	135	0.32	0.23	0.24	0.18

**Table 3:** Regression results for the direct effect and moderation effect.

	Promotion-Oriented Cognitive Crafting	Risk-Taking Behavior	Creativity
Variables	*β* (*SE*)	*β* (*SE*)	*β* (*SE*)
Age	0.001 (0.004)	0.01 (0.01)	−0.001 (0.01)
Gender	−0.07 (0.05)	0.05 (0.07)	0.02 (0.077)
Education	−0.01 (0.03)	−0.004 (0.04)	−0.01 (0.04)
Tenure	0.01 (0.01)	−0.02 (0.01)	−0.004 (0.01)
Positive workplace gossip	0.16 (0.02) ***		
Internal locus of control	0−0.02 (0.03)		
Positive workplace gossip about self × internal locus of control	0.21 (0.03) ***		
Promotion-oriented cognitive crafting		0.43 (0.07) ***	
Risk-taking behavior			0.38 (0.05) ***

Note. *N* = 463. *** *p* < 0.001. Unstandardized coefficients are reported.

**Table 4:** Conditional indirect effects.

Moderated Mediation Effects via Promotion-Oriented Cognitive Crafting and Risk-Taking Behavior	Creativity			
	Estimate	*SE*	Bootstrap LL CI	Bootstrap UL CI
Mean −1 SD (internal locus of control)	−0.01	0.01	−0.02	0.01
Mean (internal locus of control)	0.03	0.01	0.02	0.04
Mean + 1 SD (internal locus of control)	0.06	0.01	0.04	0.09

## Data Availability

The raw data supporting the conclusions of this article will be made available by the authors without undue reservation.

## Appendix A

**Items**

**Positive workplace gossip** (Chandra & Robinson, 2009)

1. In the past six months, others (e.g., coworkers and/or supervisors) communicated positive information about me in the workplace.
2. In the past six months, others (e.g., coworkers and/or supervisors) held positive discussions about me in the workplace.
3. In the past six months, others (e.g., coworkers and/or supervisors) gave positive evaluations of me in the workplace.

1. 在过去的六个月里，其他人（例如，同事和/或主管）在工作场所传播关于我的积极信息。
2. 在过去的六个月里，其他人（例如，同事和/或主管）在工作场所就我进行了积极的讨论。
3. 在过去的六个月里，其他人（例如，同事和/或主管）在工作场所对我的表现给予了积极的评价。

**Internal locus of control** (Hsiao et al., 2016)

1. I believe that effort can alter my life.
2. I believe that my level of involvement determines the resulting outcome.
3. I typically apply a proactive approach to my life.

1. 我相信努力可以改变我的生活。
2. 我相信我的参与程度决定了最终的结果。
3. 我通常以积极主动的方式对待我的生活。

**Promotion-oriented cognitive crafting** (Bindl et al., 2019)

1. I tried to think of my job as a whole, rather than as separate tasks.
2. I thought about how my job contributed to the organization’s goals.
3. I thought about new ways of viewing my overall job.
4. I thought about ways in which my job as a whole contributed to society.

1. 我试图将我的工作视为一个整体，而不是将其视为独立的任务。
2. 我思考我的工作如何为组织的目标做出贡献。
3. 我思考看待我整体工作的新方法。
4. 我思考我的整体工作如何为社会做出贡献。

**Risk-taking behavior** (Neves & Eisenberger, 2014)

1. This employee willingly accepts tasks having a high likelihood of problems.
2. This employee puts himself or herself in a position of risk to help this organization.
3. This employee tells me when he/she has made a mistake that he/she could easily hide.
4. This employee values taking a chance on new products, services, or procedures.

1. 这位员工愿意接受很有可能出现问题的任务。
2. 这位员工为了帮助组织，会将自己置于风险之中。
3. 这位员工会告诉我他/她犯了一个很容易隐藏的错误。
4. 这位员工重视尝试新产品、服务或程序的机会。

**Creativity** (Baer & Oldham, 2006)

1. This employee suggests many creative ideas that might improve working conditions.
2. This employee often comes up with creative solutions to problems at work.
3. This employee suggests new ways of performing work tasks.
4. This employee is a good source of creative ideas.

1. 这位员工提出了许多可能改善工作条件的创造性想法。
2. 这位员工经常提出解决工作问题的创造性方案。
3. 这位员工提出了执行工作任务的新方法。
4. 这位员工是创造性想法的良好来源。
